# Contribution of Antioxidant System Components to the Long-Term Physiological and Protective Effect of Salicylic Acid on Wheat under Salinity Conditions

**DOI:** 10.3390/plants13111569

**Published:** 2024-06-06

**Authors:** Dilara Maslennikova, Inna Knyazeva, Oksana Vershinina, Andrey Titenkov, Oksana Lastochkina

**Affiliations:** 1 Ufa Federal Research Center, Institute of Biochemistry and Genetics, 450054 Ufa, Russia; oksana.lastochkina@ufaras.ru; 2 Federal State Budgetary Scientific Institution «Federal Scientific Agroengineering Center VIM», 109428 Moscow, Russia; knyazewa.inna@yandex.ru (I.K.); vershinina.oks@yandex.ru (O.V.); tiandr1996@yandex.ru (A.T.)

**Keywords:** *Triticum aestivum* L., salicylic acid, salinity, antioxidant system, photosynthetic pigments, grain yield, grain amino acid profile

## Abstract

Salicylic acid (SA) plays a crucial role in regulating plant growth and development and mitigating the negative effects of various stresses, including salinity. In this study, the effect of 50 μM SA on the physiological and biochemical parameters of wheat plants under normal and stress conditions was investigated. The results showed that on the 28th day of the growing season, SA pretreatment continued to stimulate the growth of wheat plants. This was evident through an increase in shoot length and leaf area, with the regulation of leaf blade width playing a significant role in this effect. Additionally, SA improved photosynthesis by increasing the content of chlorophyll a (Chl a) and carotenoids (Car), resulting in an increased TAP (total amount of pigments) index in the leaves. Furthermore, SA treatment led to a balanced increase in the levels of reduced glutathione (GSH) and oxidized glutathione (GSSG) in the leaves, accompanied by a slight but significant accumulation of ascorbic acid (ASA), hydrogen peroxide (H_2_O_2_), proline, and the activation of glutathione reductase (GR) and ascorbate peroxidase (APX). Exposure to salt stress for 28 days resulted in a reduction in length and leaf area, photosynthetic pigments, and GSH and ASA content in wheat leaves. It also led to the accumulation of H_2_O_2_ and proline and significant activation of GR and APX. However, SA pretreatment exhibited a long-term growth-stimulating and protective effect under stress conditions. It significantly mitigated the negative impacts of salinity on leaf area, photosynthetic pigments, proline accumulation, lipid peroxidation, and H_2_O_2_. Furthermore, SA reduced the salinity-induced depletion of GSH and ASA levels, which was associated with the modulation of GR and APX activities. In small-scale field experiments conducted under natural growing conditions, pre-sowing seed treatment with 50 μM SA improved the main indicators of grain yield and increased the content of essential amino acids in wheat grains. Thus, SA pretreatment can be considered an effective approach for providing prolonged protection to wheat plants under salinity and improving grain yield and quality.

## 1. Introduction

The most widely cultivated grain on the planet is wheat (*Triticum aestivum* L.). It contributes to nearly a fifth of the total calories consumed by people and has the highest protein content among all grains [1,2,3]. The human population is growing exponentially and requires a substantial increase in global agricultural production. In order to feed the projected 10 billion people by 2050, wheat yield needs to be improved by 60–70 percent while preserving or enhancing its nutritional qualities [1,4]. According to Rudoy and co-authors, the data suggest that by 2030, wheat will continue to hold the largest share (60.4%) in the production structure of crop production in Russia [5]. Therefore, it is crucial to focus on enhancing wheat quality and yield in light of the impending global salinization during this stage of crop production development.

Salinity is anticipated to cause losses of more than 50 percent in agriculture by 2050, severely impeding plant growth and development and impacting wheat yields [6]. Approximately one-third of agricultural land globally (~950 million hectares, including 250 million hectares of irrigated land) is affected by salt stress [7,8]. It is important to consider that plants have a fixed lifestyle and must rapidly adapt their response mechanisms and metabolism to changes in their environment. Salinity often leads to an excessive accumulation of reactive oxygen species (ROS), which can interact with vital components of plant cells and cause oxidative damage, including DNA damage, lipid peroxidation, enzyme inactivation, protein oxidation, and disruptions in hormone and nutrient levels [9,10]. Salinity also has detrimental effects on the phenological development of wheat, including leaf number, leaf expansion rate, and a reduction in leaf and root surface area, resulting in decreased nutrient uptake by roots [8] and altered root/shoot ratio [11], ultimately leading to reduced photosynthetic activity, biomass production, and yield [8,9,12].

The antioxidant system components play a crucial role in such adaptation [1]. The assessment of the involvement of the ascorbate–glutathione complex components in these processes has gained significant attention [10,13,14,15]. Ascorbate (ASA) and glutathione (GSH) are important molecules that regulate cell division, thereby contributing to growth stimulation [15]. Moreover, they play a vital role in maintaining the antioxidant status and protective functions [10,16]. The ASA-GSH cycle is unique, as both ASA and GSH work together to detoxify H_2_O_2_, while simultaneously regenerating each other [14,15]. Ascorbate peroxidase (APX) converts H_2_O_2_ into water using ASA as an electron donor, which is then converted to monodehydroascorbate (MDHA). MDHA is then regenerated back to ASA by the activity of MDHAR, with a portion spontaneously converting to dehydroascorbate (DHA). DHA is further reduced back to ASA using GSH, resulting in the oxidation of GSH to produce GSSG. Finally, GSSG is regenerated to GSH by the activity of glutathione reductase (GR) using NADPH as the electron donor [14]. ASA and GSH possess high redox potentials, enabling interactions with various components and pathways to maintain a reduced state, with GR and APX being key enzymes in this cycle.

To enhance wheat resistance under salinity conditions, various methods are employed, including the use of hormonal growth regulators, nanoparticles, biochar, biochar enriched with mineral elements, and bacterial-based biological products [3,4,9,16,17,18]. Seed priming with substances that have growth-stimulating and protective effects is a widely used, cost-effective approach to increasing wheat productivity [9,12,14,19].

Salicylic acid (SA), a phytohormone of phenolic nature, plays a fundamental role in regulating plant growth and development. A review of the literature data confirms the effectiveness of seed priming with SA in increasing the resistance of wheat to various stresses [4,6,12,20,21,22,23,24,25,26,27,28]. In previous research, we determined that a concentration of 50 µM SA had a stimulating and protective effect on wheat plants [26]. The effectiveness of 50 µM SA was attributed to its ability to regulate the hormonal status. Specifically, soaking seeds in 50 μM SA for 3 h resulted in the accumulation of abscisic acid (ABA), indole-3-acetic acid (IAA), and cytokinins during the early stages of development (up to 5 days of ontogenesis) [26]. Furthermore, SA was found to significantly increase the endogenous SA levels in plants, which persisted for up to 6 days of the growing season and contributed to the growth-stimulating effects of the phytohormone, such as increased seed germination and leaf area index. Additionally, the physiological effect of SA on wheat involved the participation of the antioxidant system components, whereby 50 µM SA modulated the activity of GR and APX, resulting in a balanced accumulation of GSH, GSSG, and ASA [27]. This regulatory ability of SA on the state of the ASA-GSH complex was particularly effective under salinization conditions (100 mM NaCl). In these plants, SA reduced the depletion of ASA and GSH levels and induced the stress-responsive activation of GR and APX, mitigating the negative effects on plant growth. The data obtained suggest that SA can continue to exert growth-stimulating and protective effects for up to 7 days of growth [27]. These data are a prerequisite for clarifying how long and effectively priming wheat seeds with 50 µM SA has an effect on wheat metabolism. The majority of previous studies have focused on the short-term or immediate responses of plants to SA treatment, and the underlying mechanisms of long-term growth-stimulating and protective effects of SA under stress conditions remain largely unexplored.

The aim of our study was to assess the duration of the growth-promoting and protective effects of 50 µM SA on wheat under salinization conditions. We examined the physiological and biochemical parameters of wheat plants on the 28th day of the experiment. In order to demonstrate the substantial physiological impact of 50 μM SA, field experiments were conducted to evaluate key components of grain yield and the amino acid profile.

## 2. Results

### 2.1. Effects of SA on Wheat Plant Growth, Photosynthetic Pigments, and Biochemical Attributes under Controlled Conditions

The estimation of growth parameters is crucial for assessing the physiological state of plants. It serves as an indicator of the effectiveness of plant growth regulators and the impact of different stress factors.

**Figure 1:**
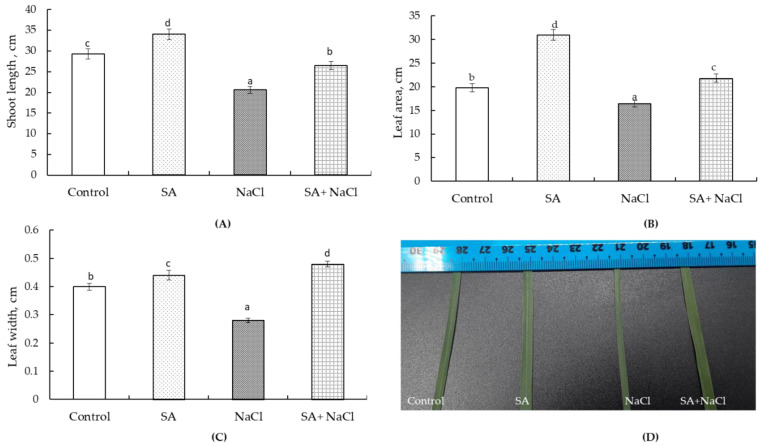
The effect of salt stress (100 mM NaCl) and pretreatment with 50 µM salicylic acid (SA) on shoot length (**A**), leaf area (**B**), and leaf width (**C**), and a visual assessment of sheet width (**D**) of wheat seedlings. All statistical differences were compared to the control group. The data represent the averages of three replications (*n* = 30) and standard errors; different letters on the top of the columns indicate statistically significant differences between the groups (*p* ≤ 0.05).

On the 28th day of the experiment, 100 mM NaCl resulted in a 1.4-fold decrease in shoot length and a 1.2-fold leaf area (Figure 1A,B). Treatment with SA increased shoot length and leaf area by 116–117% compared to the control under normal conditions. However, under stress conditions, the shoot length was 1.1 times lower and the leaf area was 1.1 above of these plants to the control level (Figure 1A,B).

It was revealed that the width of the leaf plate significantly influenced the leaf area. SA treatment led to a 1.1-fold increase in this parameter compared to the control. On the other hand, 100 mM NaCl caused a 1.3-fold reduction in leaf width. During SA treatment, this indicator was 1.2 times higher than the control value (Figure 1C). This effect of SA is confirmed by visual assessment of the width of wheat leaves (Figure 1D).

**Figure 2:**
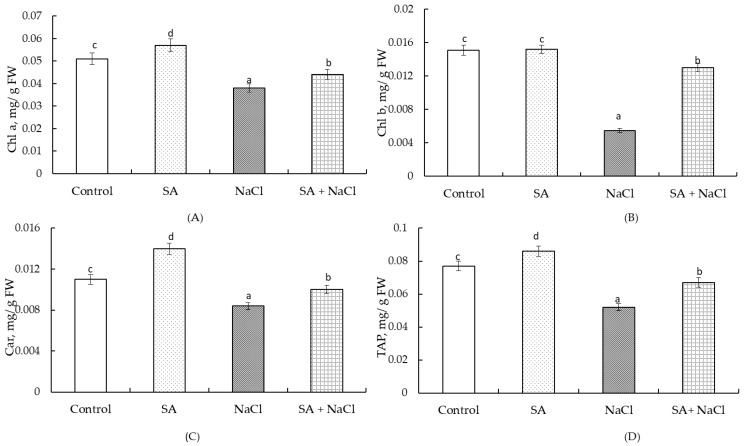
The effect of salt-stress (100 мM NaCl) and pretreatment with 50 µM salicylic acid (SA) on the content of chlorophyll *a* (Chl *a*) (**A**), chlorophyll *b* (Chl *b*) (**B**), carotenoids (Car) (**C**), and the total amount of photosynthetic pigments (TAP) (**D**) in wheat seedling leaves. All statistical differences were compared to the control group. The data represent the averages from three replications and standard errors. Different letters indicate statistically significant differences between the groups (*p* ≤ 0.05).

Our findings demonstrate that SA has an impact on the content of photosynthetic pigments in wheat leaves under both normal and salinity conditions. Pretreatment of seeds with 50 μM SA resulted in a 14% increase in chlorophyll *a* and a 27% increase in carotenoids in leaves under normal growth conditions (Figure 2A,C). The levels of chlorophyll *b* in these plants remained within the control range (Figure 2B). Additionally, these plants exhibited a 12% increase in the total amount of photosynthetic pigments (TAP) compared to the control (Figure 2D).

Under stress conditions, there was a decrease in the content of chlorophyll *a* by 25%, chlorophyll *b* by 274%, and carotenoids by 30%. Consequently, the TAP decreased by 48% compared to the control level (Figure 2D).

Under salinity conditions, SA treatment reduced the depletion of photosynthetic pigments induced by stress. The content of chlorophyll *a* and chlorophyll *b* was 14% lower, while carotenoids were 10% lower compared to the control values (Figure 2A–C). As a result, the TAP decreased by 13% relative to the control level (Figure 2D).

Salt stress caused a significant depletion (almost 2 times) of GSH and ASA. This depletion was accompanied by an accumulation of GSSG (1.7 times) and H_2_O_2_ (1.6 times), leading to a significant increase in MDA concentration and a 3-fold decrease in the GSH/GSSG ratio. Additionally, there was a 1.9-fold increase in GR activity and a 1.8-fold increase in APX activity. The proline content in these plants was 1.4 times higher compared to the control (Table 1).

Under normal conditions, pretreatment with SA resulted in a balanced accumulation of GSH and GSSG, maintaining the GSH/GSSG ratio at the control level. Additionally, seed soaking with SA led to an insignificant but reliable increase in the content of ASA, proline, and H_2_O_2_. The activity of GR and APX in these plants was 109% higher compared to the control (Table 1).

### 2.2. Effect of SA on Yield Components, Grain Amino Acid Profiles, and Other Quality Parameters of Wheat under Small-Scale Field Conditions

Table 2 represents the results demonstrating that the pretreatment of seeds with SA increased the length of shoots (above-ground part of plants) by 1.1 times, the length of spikes by 1.3 times, and the weight of grains by 115% compared to the control group.

Data on the effect of 50 µM SA on the composition of individual amino acids, carbohydrate content (mg 100^−1^ dry weight), and raw fat (%) of wheat grain can be found in Table 3. SA pretreatment had an influence on the amino acid composition of the grains.

SA treatment improved the content of all essential amino acids in the wheat grain, increasing it by 110% compared to the control group. Additionally, SA increased the content of non-essential amino acids, except for aspartic acid + asparagine and glutamic acid. However, SA did not have a significant effect on the carbohydrate content (fructose, glucose, and sucrose) in the grain. On the other hand, SA increased the raw fat content by 125% relative to the control values. These novel findings highlight the potential of SA pretreatment as an effective approach for providing prolonged protection to wheat plants under salinity and improving grain yield and quality.

## 3. Discussion

The initial goal of our study was to evaluate whether the growth-stimulating effect of SA lasts up to 28 days of wheat ontogenesis. The results showed that seed priming with 50 μM SA for 3 h stimulated plant growth (Figure 1A). There is evidence in the literature that pre-sowing seed soaking with SA has a prolonged impact on wheat plant growth. For instance, El-Hawary and co-authors observed that soaking wheat seeds in SA (1.45 mM) for 8 h stimulated plant growth (leaf area index) for up to 95 days [22]. Similarly, soaking wheat seeds for 15 h in 10 and 20 mM SA solutions had a long-term growth-stimulating effect, lasting up to 50 days of development [21]. Similar to our results, Azeem and co-authors found a growth-stimulating effect of SA on the 28th day of growth for wheat seeds treated with 0.5 mM and 1 mM SA for 12 h [24], while Alam et al. observed a similar effect on the 30th day when seeds were treated in with 0.5 mM SA for 12 h [23]. The novel aspects of our study include the investigation of the SA-mediated regulation of leaf blade width and its impact on leaf area, the detailed analysis of photosynthetic pigments and antioxidant metabolism, and the evaluation of the field-level impacts of SA pretreatment on wheat productivity and grain quality. The findings of the presented study are expected to provide valuable insights into the mechanisms by which SA can offer prolonged protection to wheat plants under salinity and have important implications for the development of sustainable agricultural practices. We discovered that SA contributes significantly to the increase in the area of wheat leaf blades (Figure 1B) by promoting their width expansion (Figure 1C,D). This ability of SA to regulate leaf growth may explain its mitigating effect on leaf width damage caused by salinization. This regulatory effect is likely associated with SA’s ability to modulate cell division and expansion, as evidenced by higher expression of the cell cycle G1/S transition regulator cyclin D (CYC3) and increased endoreduplication concentrations in NahG transgenic plants, resulting in larger cells [29]. Another mechanism through which SA enhances plant growth is via the accumulation of tryptophan biosynthesis, which is responsible for accelerated growth rates. These findings confirm the important role of SA in promoting the growth of wheat plants, both in the presence and absence of NaCl, which aligns with previous research conducted on maize following SA treatment [30].

Soaking seeds has been found to modulate the photosynthetic activity of plants [31]. Our study revealed an increase in the content of chlorophyll *a*, carotenoids (Figure 2A,B), and the TAP (Figure 2D) on the 28th day of growth in plants primed with SA. A similar accumulation of chlorophyll *a* and carotenoids were observed by Maqsood et al. on the 50th day of the growing season [21]. This increase in photosynthetic pigments improves the efficiency of photosynthetic processes in the leaves [32,33,34] and enhances the antioxidant status of the plants [34,35]. In saline soil conditions, photosynthetic pigments were reduced, as observed in our study (Figure 2) and reported in the literature [21,22,23,24]. However, pretreatment with SA protects the photosynthetic apparatus of wheat leaves from the negative effects of stress-induced oxidative stress and enhances the efficiency of photosynthesis (Figure 2).

The application of exogenous SA has been shown to enhance the effectiveness of antioxidants in various biological pathways [23]. We believe that the long-term growth-stimulating effect of SA is associated with its ability to maintain increased levels of GSH and GSSG, without affecting the GSH/GSSG ratio. The content of ASA in these plants is higher than in the control group. Furthermore, the increase in proline content in these plants contributes to their enhanced antioxidant status (Table 1). SA regulates proline content in wheat in different ways, with a decrease observed on the 30th day of the growing season [23] and accumulation on the 50th day [21] and 90th day of the growing season [28]. It is worth noting the positive effect of exogenously applied GSH, ASA, and proline in enhancing plant resistance to stress. This is likely due to the fact that these antioxidants do not exhibit toxic effects, as they are efficiently catabolized in plants [36,37].

In addition to the accumulation of proline, we found that SA helps to maintain the level of H_2_O_2_ and the activity of GR and APX in the plants. There is evidence in the literature confirming the positive activation of antioxidant enzymes on both the 30th [23] and 50th [21] days of plant growth. Moreover, H_2_O_2_ acts as a signaling molecule in this context, as it has been demonstrated that SA positively regulates its accumulation [27,38]. H_2_O_2_ is a key molecule involved in a wide range of physiological processes, such as growth, development, flowering, and photosynthesis, as well as protective reactions under stress [39,40].

This indicates in favor of the fact that in our case, pre-sowing seed soaking with 50 µm SA positively regulates the antioxidant status in wheat leaves. This ability is clearly demonstrated when analyzing all the studied parameters under 100 mM NaCl. Under stress conditions, SA treatment effectively prevents GSH and ASA depletion while inhibiting significant GSSG accumulation. Perhaps, the observed effect of SA in this context may be attributed to its ability to regulate the expression of genes associated with the ascorbate–glutathione complex during drought conditions [16].

Thus, SA is able to regulate the state of the antioxidant system for a sufficiently long time (28th day), which manifests itself in reducing the damaging effect of salinity on the growth (Figure 1) and integrity of membrane structures (Table 1). It should be noted that the growth-stimulating effect of SA is prolonged, as reflected in the assessment of wheat yield under natural growing conditions, which is confirmed by the literature data [4,12,20,21,22] and our results (Table 2). Experiments to evaluate SA on the state of the antioxidant system were limited to 28 days of vegetation. This is due to the fact that we did not detect a significant effect of SA on this system at later stages of ontogeny. Importantly, SA not only increases wheat yield but also improves grain quality. Seed storage proteins play a crucial role during the early stages of plant growth. They are necessary for the respiratory metabolism of seeds, germination and growth of seedlings, and regulation of physiological and biochemical reactions and metabolic processes [41]. Along with this, proteins are essential components of human nutrition and are vital for normal growth and development. According to the World Health Organization, half of hunger is caused by protein deficiency. Amino acids are the building blocks of all proteins. There are non-essential and essential amino acids. Essential amino acid deficiency leads to serious human diseases [42]. It is worth mentioning that there is limited literature on the impact of SA on the amino acid composition of wheat grains. Aldesuquy H. found that pre-sowing seed soaking with 50 µM SA resulted in the accumulation of certain essential amino acids such as valine, leucine, phenylalanine, threonine, and histidine [25]. In our study, we observed an increase in the content of all essential (valine, leucine + isoleucine, methionine, tryptophan, phenylalanine, lysine, threonine, histidine) and some non-essential (arginine, serine, proline, glycine, alanine, cysteine) amino acids (Table 3). Hence, the increased content of all essential amino acids in the grain of SA-treated plants, as discovered in our research, contributes to its improved nutritional value.

## 4. Materials and Methods

### 4.1. Plant Material and Seed Treatments

The experiments were carried out on soft spring wheat (*Triticum aestivum* L., BBAADD 2n = 42, “Salavat Yulaev”). This wheat was obtained by taking into account the peculiarities of the climate of the Republic of Bashkortostan (RB), Russia [43]. Wheat seeds were obtained from Chishminsky Breeding Station, Ufa Federal Research Centre, Russian Academy of Sciences (Ufa, RB, Russia). The seeds were sterilized by immersing them in 96% ethanol for 60 s. Subsequently, they were washed with sterile water until the smell of alcohol disappeared. To soak the seeds, a solution of salicylic acid [SA: (C_7_H_6_O_3_)] was utilized [26]. The seeds were kept in a solution of 50 μM SA or water (Control) for 3 h [26,27]. Following the soaking process, the seeds were air dried prior to planting.

### 4.2. Design of Experiments and Growth Conditions

In the first set of experiments, seeds pretreated and untreated with 50 μM SA (previously selected concentration [26]) were planted in plastic pots filled with 6 kg of commercially available «Universal» soil (“Alliance”, Berezovsky, Russia). The soil used had an optimal content NPK ratio (total nitrogen (NO_3_ + NH_4_) ≥ 1%, total phosphorus (P_2_O_5_) ≥ 1%, total potassium (K_2_O) ≥ 0.5%), pH ranging from 6.2 to 6.5, and a moisture level of 65–68%. Additionally, we assessed the soil pH and humidity using a digital soil analyzer, the KC-300 (Qingdao Tlead International Co., Ltd., Qingdao, China). To establish the required salinity level, the soil was carefully prepared. It was crushed, dried, and then saturated with a solution of 100 mM NaCl [44]. Some plants were planted in soil saturated with water, representing normal growth conditions, while others were planted in soil saturated with 100 mM NaCl, representing stressed growth conditions. Each pot contained 10 plants with 4 replicates for each group of experimental plants. On the 28th day of ontogenesis, physiological and biochemical parameters were assessed in wheat plants. These parameters included length shoot and leaf area, proline content, photosynthetic pigments, hydrogen peroxide (H_2_O_2_), malondialdehyde (MDA), ascorbic acids (ASAs), reduced glutathione (GSH) and its oxidized form (GSSG), and the activities of key enzymes, including glutathione reductase (GR) and ascorbate peroxidase (APX).

In another experimental setting, pretreated and untreated SA seeds were cultivated under natural growing conditions. Small-scale field experiments were performed in 2021–2022 at the rural settlement “Alkinsky village council” (Chishminsky district, Bashkortostan Republic, Russia) at 54°34′ N and 55°22′ E and an elevation of 116 m above sea level. The experimental field’s soil characteristics included leached chernozem (pH 5.5), the content of humus—8.4%, Hg—5.50 mg-EQ/100 g soil, pH_KCl_—5.9, exchangeable K—30 mg/100 g soil, and mobile P—23 mg/100 g soil. The pretreated and untreated seeds were sown at a depth of 4–5 cm, with a distance of 3 cm between the plants and 8 cm between the rows. Two plots measuring 1 m^2^ were allocated for each group of experimental plants. Crop yields were measured in both 2021 and 2022. The climate characteristics during the growing season were as follows: the sum of rainfall from May to August was approximately 80 mm in 2021 and 172 mm in 2022. The average air temperatures in May, June, July, and August were, respectively, 17.8 °C, 21 °C, 21 °C, and 22.8 °C in 2021, and 10.9 °C, 16.7 °C, 21 °C, and 21 °C in 2022. The highest temperature recorded in 2021 was 38 °C, while in 2022, it was 24.8 °C. The lowest temperature recorded in 2021 was 2 °C, and in 2022, it was 6 °C. Yield component parameters and grain analysis were conducted on the 90th–92nd day of ontogeny, according to the variety of characteristics [43].

### 4.3. Analysis of Growth Indicators of Shoots

Shoot length was measured using a meter scale [23]. The measurement of the leaf area was performed using the Quarry and Jones equation [45] as follows:

Leaf area = (Leaf length × Leaf width) × 0.75

The leaf length was determined at the junction of the leaf blade and the petiole. The width of the leaf was measured as the distance between two points on the edge of the leaf blade, perpendicular to the axis of the leaf length.

### 4.4. Chlorophyll and Carotenoid Content

Equal amounts of fresh wheat leaves (0.1 g) were homogenized in 10 mL of 80% acetone. The homogenate was then placed in the dark for 40 h and subsequently filtered. The optical density of the prepared extracts was measured using an EnSpire Model 2300 Multilabel Microplate Reader (PerkinElmer, Waltham, MA, USA) at specific wavelengths of 663 nm for chlorophyll *a*, 646 nm for chlorophyll *b*, and 470 nm for carotenoids. The results are expressed as milligrams per gram of fresh weight (mg/g FW) [46]. The total amount of photosynthetic pigments (TAP) was calculated as the sum of chlorophyll *a*, chlorophyll *b*, and carotenoids.

### 4.5. Measurement of Reduced Glutathione (GSH) and Oxidized Glutathione (GSSG) Contents

The levels of glutathione (GSH) that needed to be measured were determined using o-phthalaldehyde (Sigma-Aldrich, St. Louis, MO, USA) as a fluorescent reagent. To prevent GSH autooxidation and the derivatization of GSH, N-ethylmaleimide (Sigma-Aldrich, St. Louis, MO, USA) was used for the derivatization of GSH [47,48]. The GSH/GSSG ratio, which indicates plant redox status, was determined as the ratio of total glutathione (GSH + GSSG) and oxidized glutathione (GSSG). The content of GSH and GSSG was expressed in μmoL/mg protein.

### 4.6. Antioxidant Enzyme Activity Analysis

The activity of glutathione reductase (GR,EC:1.6.4.2) was measured following the method described in [47]. The reaction was initiated by adding GSSG, and the decrease in absorbance at 340 nm was recorded for 1 min. The activity was calculated using an extinction coefficient of 6.2 mM ^−1^cm^−1^ and expressed as nmoL/mg protein min.

The activity of ascorbate peroxidase (APX,EC:1.11.1.11) was assayed according to the method described in [47]. The reaction buffer solution contained K-P buffer (pH 7.0), ASA, H_2_O_2_, and EDTA. The decrease in absorbance at 290 nm was monitored for 1 min, and the activity was calculated using an extinction coefficient of 2.8 mM ^−1^cm^−1^. The results were expressed as µmoL ascorbate oxidized/mg min protein.

Protein concentrations were determined using the Bradford method [49].

### 4.7. Ascorbic Acid (ASA) Content Assay

The analysis of ascorbic acid (ASA) content was performed using the titration method, as described in detail by [47]. The ASA content was determined in whole plants and expressed as mg% FW.

### 4.8. Quantification of Oxidative Stress Indices

The products of lipid peroxidation in wheat were determined using the method described by [50], which involves measuring the concentration of reactive substances, primarily malondialdehyde (MDA), using thiobarbituric acid. The MDA content was expressed as nM/g FW.

To quantify H_2_O_2_, the xylenol orange method in the presence of Fe^2+^ was employed, following the method outlined in [47]. The absorbance was measured at 560 nm, and the H_2_O_2_ content was expressed as µmoL/g FW.

### 4.9. Yield Components

At harvest, several measurements were taken on the wheat plants. These include shoot length (the length of the above-ground part of the plant) (cm), spike length (cm), and 1000 grain weight (g) [51].

### 4.10. Grain Quality Analysis (Amino Acids, Carbohydrates, and Raw Fat)

The mass fractions of amino acids in the samples were determined through acid and alkaline hydrolysis, converting them into free forms and obtaining phenylisothiocarbamil derivatives. The separation and quantitative determination of amino acids were carried out by capillary electrophoresis with the “Kapel-205” device (Lumex-Marketing LLC, St. Petersburg, Russia) according to [52].

The mass fraction of carbohydrates was determined by preparing a sample and separating and identifying its components using capillary electrophoresis. Briefly, a crushed and homogenized sample was centrifuged for 5 min at 5000 rpm, and the resulting supernatant was analyzed using the “Kapel-205” capillary electrophoresis device (Lumex-Marketing LLC, St. Petersburg, Russia), following the certified method for measuring the mass fraction of mono- and disaccharides, as regulated by the document M 04-92-2020 “Food products, food raw materials, feed, and food additives”.

The mass fraction of raw fat was determined according to [53], for this purpose, fat was extracted from the sample using petroleum ether in the Soxhlet apparatus. The solvent was then removed, and the remaining residue was weighed. 

### 4.11. Statistical Analysis

All physiological and biochemical analyses were carried out in three biological and three analytical replicates. Field experiments were performed twice, and the crop structure was analyzed in three biological replicates. The content of amino acids, fat, and carbohydrates was analyzed in two biological and three chemical repeats. Experimental data were presented as means ± SE, which were calculated for all treatments using MS Excel. The significance of differences was evaluated using ANOVA followed by Duncan’s test (*p* ≤ 0.05) with STATISTICA 10.0 software.

## 5. Conclusions

This study was aimed at studying the effectiveness of seed priming with 50 μM SA under long-term experimental conditions. It was discovered that 50 μM SA exerts a stimulating and protective effect on wheat plants under salinity. SA demonstrates the ability to positively regulate the state of the components of the ascorbate–glutathione complex for up to 28 days of the growing season, thereby increasing the antioxidant status of the plants. The results demonstrated that SA contributes to a slight but significant increase in the accumulation of ASA, GSH, and H_2_O_2_. Furthermore, it activates GR and APX while maintaining elevated levels of chlorophyll *a*, carotenoids, and proline. These SA-induced reactions in wheat significantly mitigate the damage caused by stress on the antioxidant system components under 100 mM NaCl conditions. Additionally, we propose that the prolonged growth-stimulating effect is manifested in increased wheat yield and improved nutritional value of the grains. Particularly, pre-sowing seed treatment with SA resulted in the formation of wheat grains with increased contents of essential (valine, leucine + isoleucine, methionine, tryptophan, phenylalanine, lysine, threonine, histidine) and non-essential (arginine, serine, proline, glycine, alanine, cysteine) amino acids, whereas SA did not have a significant effect on the grains’ carbohydrate content (fructose, glucose, and sucrose) but increased the raw fat content.

## Figures and Tables

**Table 1:** Influence of 50 µM salicylic acid (SA) on the content of GSH, GSSG, the GSH/GSSG ratio, ASA, MDA, H_2_O_2,_ proline, and the state of antioxidant enzymes, GR and APX, in the leaves of wheat seedlings under salinity stress (100 mM NaCl).

Treatment	GSH, µmoL/mg Protein	GSSG, µmoL/mg Protein	GSH/ GSSG Ratio	ASA, mg% FW	GR, nmoL/mg Protein min	APX, μmol Ascorbate Oxidized/ mg Protein min	H_2_O_2_, µmoL/g FW	Proline, µmoL/g FW	MDA, nM/g FW
Control	63 ± 2.5 ^a^	5.5 ± 0.22 ^c^	11.4 ± 0.4 ^a^	2.2 ± 0.09 ^b^	3.9 ± 0.15 ^d^	5.7 ± 0.22 ^d^	3.8 ± 0.15 ^d^	15 ± 0.52 ^d^	5.9 ± 0.22 ^c^
SA	62 ± 2.5 ^a^	5.3 ± 0.21 ^c^	11.6 ± 1.5 ^a^	2.4 ± 0.10 ^a^	4.2 ± 0.18 ^c^	6.2 ± 0.23 ^c^	4.2 ± 0.17 ^c^	17 ± 0.75 ^c^	6.1 ± 0.36 ^c^
NaCl	33 ± 1.3 ^c^	9.4 ± 0.38 ^a^	3.5 ± 0.16 ^c^	1.1 ± 0.04 ^d^	7.4 ± 0.29 ^a^	10.4 ± 0.44 ^a^	6.1 ± 0.24 ^a^	21 ± 1.2 ^a^	8.26 ± 0.32 ^a^
SA + NaCl	42 ± 1.7 ^b^	7.5 ± 0.11 ^b^	5.6 ± 0.22 ^b^	1.4 ± 0.06 ^c^	6.2 ± 0.25 ^b^	7.58 ± 0.3 ^b^	4.8 ± 0.19 ^b^	19 ± 0.76 ^b^	7.1 ± 0.29 ^b^

All statistical differences were submitted regarding the control variant. The data are averages from three SE replications; the averages with different letters are significantly different (*p* ≤ 0.05).

**Table 2:** The effect of salicylic acid (SA) pretreatment on the yield components of wheat plants. The table presented contains average data for the years 2021 and 2022.

Year	Treatments	Shoot Length (cm)	Spike Length (cm)	1000 Grain Weight (g)
2021	Control	103 ± 4.1 ^a^	9.5 ± 0.37 ^a^	38.8 ± 1.48 ^a^
	50 µM SA	116 ± 4.6 ^b^	12.2 ± 0.48 ^b^	44.9 ± 1.76 ^b^
2022	Control	109 ± 4.4 ^a^	10.9 ± 0.4 ^a^	40.3 ± 1.57 ^a^
	50 µM SA	125 ± 4.9 ^b^	13.1 ± 0.52 ^b^	46.1 ± 1.88 ^b^

All statistical differences were submitted regarding the control variant. The data are averages from three SE replications; the averages with different letters are significantly different (*p* ≤ 0.05).

**Table 3:** Effect of salicylic acid (SA) pretreatment on amino acid (AA) profiles, the content of carbohydrate (mg/100 g DW), and raw fat (%) in wheat grains.

Parameters	Control	SA
Valine	408.07 ± 16.32 ^a^	511.75 ± 20.50 ^b^
Leucine + Isoleucine	995.42 ± 39.80 ^a^	1140.39 ± 45.60 ^b^
Methionine	151.42 ± 6.04 ^a^	185.01 ± 7.4 ^b^
Tryptophan	177.81 ± 7.20 ^a^	220.61 ± 8.8 ^b^
Phenylalanine	426.43 ± 17.04 ^a^	480.31 ± 19.24 ^b^
Lysine	327.39 ± 13.08 ^a^	390.09 ± 15.6 ^b^
Threonine	315.10 ± 12.6 ^a^	346.09 ± 16.84 ^b^
Histidine	289.92 ± 11.55 ^a^	393.89 ± 12.92 ^b^
Essential AA	3091.56 ± 123.65 ^a^	3668.13 ± 146.72 ^b^
Arginine	403.78 ± 16.1 ^a^	453.93 ± 18.132 ^b^
Serine	557.33 ± 22.28 ^a^	650.28 ± 26.05 ^b^
Proline	1054.35 ± 42.15 ^a^	1270.28 ± 50.8 ^b^
Glycine	419.93 ± 16.74 ^a^	489.76 ± 19.56 ^b^
Alanine	357.47 ± 14.67 ^a^	367.49 ± 14.68 ^b^
Aspartic acid + Asparagine	401.72 ± 16.01 ^a^	422.53 ± 16.91 ^a^
Glutamic acid	3101.82 ± 124.04 ^a^	3175.39 ± 127.00 ^a^
Cysteine	262.26 ± 10.60 ^a^	289.45 ± 11.57 ᵇ
Non-essential AA	6558.66 ± 270.45 ^a^	7101.47 ± 284.76 ^b^
Total AA	9650.02 ± 386 ^a^	10767.00 ± 430.68 ^b^
Essential AA/Non-essential AA	0.47± 0.02 ^a^	0.52 ± 0.02 ^b^
Fructose	108.67 ± 4.32 ^a^	118.93 ± 4.72 ^a^
Glucose	271.23 ± 10.84 ^a^	279.94 ± 11.19 ^a^
Sucrose	1204.09 ± 48.15 ^a^	1248.32 ± 49.28 ^a^
Raw fat	2.34 ± 0.10 ^a^	2.89 ± 0.12 ^b^

All statistical differences were submitted regarding the control variant. The data are averages from three SE replications; the averages with different letters are significantly different (*p* ≤ 0.05).

## Data Availability

Data are contained within the article.
